# Prototype Learning for Medical Time Series Classification via Human–Machine Collaboration

**DOI:** 10.3390/s24082655

**Published:** 2024-04-22

**Authors:** Jia Xie, Zhu Wang, Zhiwen Yu, Yasan Ding, Bin Guo

**Affiliations:** School of Computer Science, Northwestern Polytechnical University, Xi’an 710072, China; xiejia@mail.nwpu.edu.cn (J.X.); zhiwenyu@nwpu.edu.cn (Z.Y.); dingyasan@163.com (Y.D.); guob@nwpu.edu.cn (B.G.)

**Keywords:** time series classification, prototype learning, attention mechanisms, human–machine collaboration, ECG

## Abstract

Deep neural networks must address the dual challenge of delivering high-accuracy predictions and providing user-friendly explanations. While deep models are widely used in the field of time series modeling, deciphering the core principles that govern the models’ outputs remains a significant challenge. This is crucial for fostering the development of trusted models and facilitating domain expert validation, thereby empowering users and domain experts to utilize them confidently in high-risk decision-making contexts (e.g., decision-support systems in healthcare). In this work, we put forward a deep prototype learning model that supports interpretable and manipulable modeling and classification of medical time series (i.e., ECG signal). Specifically, we first optimize the representation of single heartbeat data by employing a bidirectional long short-term memory and attention mechanism, and then construct prototypes during the training phase. The final classification outcomes (i.e., normal sinus rhythm, atrial fibrillation, and other rhythm) are determined by comparing the input with the obtained prototypes. Moreover, the proposed model presents a human–machine collaboration mechanism, allowing domain experts to refine the prototypes by integrating their expertise to further enhance the model’s performance (contrary to the human-in-the-loop paradigm, where humans primarily act as supervisors or correctors, intervening when required, our approach focuses on a human–machine collaboration, wherein both parties engage as partners, enabling more fluid and integrated interactions). The experimental outcomes presented herein delineate that, within the realm of binary classification tasks—specifically distinguishing between normal sinus rhythm and atrial fibrillation—our proposed model, albeit registering marginally lower performance in comparison to certain established baseline models such as Convolutional Neural Networks (CNNs) and bidirectional long short-term memory with attention mechanisms (Bi-LSTMAttns), evidently surpasses other contemporary state-of-the-art prototype baseline models. Moreover, it demonstrates significantly enhanced performance relative to these prototype baseline models in the context of triple classification tasks, which encompass normal sinus rhythm, atrial fibrillation, and other rhythm classifications. The proposed model manifests a commendable prediction accuracy of 0.8414, coupled with macro precision, recall, and F1-score metrics of 0.8449, 0.8224, and 0.8235, respectively, achieving both high classification accuracy as well as good interpretability.

## 1. Introduction

Time series is a popular data type, including prices in the shares market [1], the climate across different regions [2], electronic health records (EHRs) [3], etc. Along with the rapid development of artificial intelligence technologies, there has been a clear trend toward optimized decision making by modeling and analyzing such data during the past decade. For example, by developing a conceptual model of EHRs from varied views, optimal disease patterns can be identified, based on which the tendency of certain medical events can be predicted accordingly [4,5].

A great amount of studies have been conducted for time series analysis, and the widely used methods include dynamic time warping (DTW) [6], shapelets [7], and artificial neural networks [8]. Dynamic time warping first calculates the distance between two time series, then searches for an optimal match for them with different lengths or rhythms and supports their stretching and bending on the time axis [9]. Similarly, algorithms such as support vector machine [10], decision tree [11], and KNN [12] have been used on the same occasions. Meanwhile, there are also studies that adopt the ensemble approach by combining DTW with KNN or other methods, achieving better performance than each single approach [13]. Although these methods can locate important features within time series, they fail to identify the correlation and capture the dynamic dependencies among different variables.

In recent years, there has been a line of alternative research, and the shapelets approach [7] is one of the most promising approaches. Shapelets are discriminative phase-independent sub-sequences that reflect different patterns of the time series, i.e., each class corresponds to one or several shapelets. It has been shown that the shapelets-based approach outperforms the DTW-based approach, which classifies data samples based on their distances to different shapelets [14]. Moreover, shapelets themselves are intuitive since they can easily be retraced back to the original time series, providing high-value guidance for the supported decision-making system in critical areas, such as medical and hygienical fields. Although the shapelets-based approach had provided attractive performance, it is necessary to convert time series to an extensive set of patterns or sub-sequences as interested candidates by several parsing steps, and the larger matching space may lead to a relatively low classification accuracy.

In addition to traditional data mining methods, deep neural networks have also yielded promising results for the classification of time series. For instance, Lipton et al. used the Long Short-Term Memory (LSTM) architecture to recognize informative patterns from multivariate electronic health time series and classify the clinical records to 128 diagnostic categories [15]. Chauhan et al. utilized LSTM units to build a predictive model for normal or abnormal ECG signals [16]. By extracting informative patterns from all channels, Zheng et al. proposed a Multi-Channels Deep Convolutional Neural Network (MC-DCNN) model for time series classification [17]. Similarly, Liu et al. designed a novel multivariate convolutional neural network (MVCNN) architecture to extract patterns from co-evolving time series [18]. While deep learning approaches can achieve promising results and require less domain knowledge than traditional approaches, they are usually regarded as black boxes, which provide limited confidence and interpretability and thus are not suitable to critical applications, e.g., computer-aided diagnosis.

To overcome the above drawback, much effort has been devoted to the issue of “interpretability”. This line of methods can be divided into two categories [19], namely, post hoc interpretability and inherent interpretability. **Post hoc interpretability**: This approach endeavors to elucidate the decision-making mechanisms behind existing ’black-box’ models. The former category aims to unveil the decision-making process of existing black-box models. For this category, the first approach is hidden analysis [20], which uses the back propagation mechanism to propagate essential factors from the output layer to the input layer so as to deduce the importance of sample feature vectors. The second approach is model distillation [21], which constructs a smaller model to simulate the decision-making process of the original complex model and retains the accuracy at the same time. By reducing model complexity, it can help understand the trained model as a whole from the perspective of decision logics. The third one is sensitivity analysis [22], which is used to analyze the influence of each attribute of samples on the final classification results, thus providing explanations for the decision-making outcomes. However, these methods are designed to approximate the reasoning process rather than the real decision-making process. Thus, there may exist unfaithfulness and an inconsistent understanding of the inner-working mechanisms of sequence models [23]. The last approach is the attention mechanism [24], which employs attention weights to directly reflect the interested sub-sequences during the decision-making process of models. By mimicking physician practice, the attention mechanism is able to focus on a small portion of useful high-dimensional sequence variables and achieve both high accuracy and good interpretability [25]. Nevertheless, even though user-friendly explanations can be obtained, the attention weights are not always trustworthy [26]. In addition, attention-based methods are usually designed for domain experts (e.g., clinicians), and maybe provide unintelligible results for novice users [27]. Given such limitations, the attention mechanism is not so feasible for some practical applications [28].

Compared to post hoc interpretability models [29], **Inherent interpretability:** embeds explanation capabilities directly within the model’s architecture, offering more authentic and understandable outputs [30]. Specifically, prototype learning is one of the methods for developing such models [31]. Inspired by case-based reasoning, the prototype learning method gives a predictable conclusion for the unknown input by comparing it with a few representative cases (i.e., prototypes), e.g., exemplar patients from a cohort. The process is analogous to how doctors perform diagnosis and prescribe medications for a new patient by referring to their experiences with previous similar observations and deducing rational treatment accordingly. From an interpretable perspective, prototypes provide a more intuitive method based on visible phenotypes in time series; thus, even a novice can comprehend how the model has reached a certain conclusion, as long as they are able to understand the similarity between an input and a collection of prototypes. Such reasoning logic is widely used in nearly all existing prototype-based models [32,33]. For instance, the ProSeNet model [19] classifies each ECG event into a corresponding group based on the fact that it shares similar cardiac morphology with other explainable phenotypes, and deduces a decision by fusing the similarity. And unlike the above-mentioned shapelets which focus on identifying key sub-sequences within the time series that are strongly indicative of certain classes, providing a model based on the identification of these critical features, prototype learning, conversely, relies on the comparison of entire instances to a curated set of exemplars, offering a broader case-based understanding. However, to improve the model’s performance, the majority of existing studies choose to generate a large number of prototypes that is far greater than the number of classes, making it difficult for nonprofessional users to comprehend the obtained decisions [31].

To address the shortcoming of existing studies, in this study, we explore prototype learning and attention mechanisms to develop a deep sequence network with the fusion of human–machine intelligence (PahNet). Specifically, PahNet combines Recurrent Neural Networks (RNNs) with prototype learning in a novel framework which is designed for time series data analysis. Here, RNNs are not merely used for feature extraction; they are intricately optimized to enhance the detection of temporal patterns essential for the dynamic refinement of prototypes, allowing for a more accurate classification of unknown samples based on their temporal similarity to these enhanced prototypes. To ensure better interpretability, we design a user-friendly human–machine collaboration mechanism for fine tuning PahNet, allowing domain experts without any technical knowledge to incorporate their intuition and experience into the model by manually refining the prototypes. Moreover, we put forward a prototype quantity control method to reduce the overall number of prototypes. In particular, the prototype quantity control method operates through two main phases: generation and pruning, both guided by expert feedback. During the generation phase, a conservative approach is adopted to create a foundational set of prototypes that capture key data characteristics. Experts then guide the introduction of additional prototypes, ensuring they add value in terms of enhancing model accuracy or interpretability. In the pruning phase, we evaluate the contribution of each prototype, removing those deemed less informative. This not only streamlines the model, making it more efficient, but also simplifies the decision-making process, enhancing interpretability for nontechnical domain experts.

In this research, PahNet is employed to achieve the interpretable and adaptable classification of medical time series data, such as ECG signals in the dataset of the PhysioNet/Computing in Cardiology (CinC) Challenge 2017 [34]. By initially refining single heartbeat data representation through bidirectional long short-term memory and attention mechanisms, we proceed to generate prototypes within the training phase. Classification for conditions like normal sinus rhythm, atrial fibrillation, and other rhythms is achieved by matching inputs against these prototypes. Experimental results show that while our model slightly underperforms against certain benchmarks like CNN and Bi-LSTM with attention in binary classification tasks, it significantly outperforms existing state-of-the-art prototype models, especially in more complex triple classification scenarios, highlighting its efficacy and potential in medical time series analysis.

In general, the contributions of our work are as follows:

- To achieve both high accuracy and good interpretability, we propose PahNet to model time series by exploring prototype learning and attention mechanisms. In particular, attentional LSTM is used to extract high-quality latent features (e.g., the absence or irregularity of P waves, or the irregular rhythm that lacks a consistent pattern), based on which a set of prototypes is obtained for accurate and interpretable classification.
- A human–machine collaboration mechanism is designed to refine PahNet. Specifically, domain experts without any technical knowledge (e.g., physicians) are allowed to modify the extracted prototypes, ensuring that the model is consistent with their intellectual insights and professional considerations.
- Experimental results on a real-world dataset indicate that the proposed model significantly outperforms state-of-the-art baselines.

The remainder of this paper is organized as follows. In Section 2, we review the related work. Section 3 describes materials and the proposed PahNet in details, followed by the evaluation results in Section 4. We conclude the paper and discuss our findings in Section 5. In Section 6, we look forward to the prospects of the future research work.

## 2. Related Work

### 2.1. Traditional Approaches for Time Series Analysis

A variety of traditional methods have been applied to the analysis of time series, which can be divided into three categories, including dynamic time warping [6], time series shapelets [7], and bag of patterns [35]. DTW measures the distance between different time series, especially for those that have different rhythms or lengths [9]. For example, Wan et al. [36] introduced a clustering method based on DTW to calculate the similarity among different data recordings, which is able to retain the nature of sequence information during the clustering process. Similarly, Li et al. [37] proposed a novel clustering approach by using DTW, which is used to generate a fuzzy membership matrix to calculate the overall similarity of time series. However, DTW is more susceptible to noise [38] and may generate perceptually nonsensible alignments [39].

To address the limitations of DTW-based methods, one line of studies propose to extract short and representative patterns from time series. For instance, Ye et al. [38] introduced new time series shapelets, which can capture discriminative patterns by comparing a small subsection of time series and provide interpretable results. Similarly, Wang et al. [40] learned shapelets from data records to detect abnormality surgery objects. To obtain short patterns from time series, an alternative approach is based on the bag-of-patterns (BOP), which employs a sliding window to divide time series into a bag of sub-sequences and transforms them into symbolic patterns [35]. Liang et al. [41] introduced an approach named Hybrid Bag-Of-Patterns (HBOP), which integrates with a discretization algorithm to transform each sub-sequence into a symbolic string while maintaining the linear complexity. Based on a bag-of-features representation, Baydogan et al. [42] proposed to extract multiple sub-sequences from random locations with random lengths and provide efficient representation for the classification of time series.

While DTW, shapelets, and BOP are established methods for time series analysis, they necessitate significant domain expertise to segment time series into extensive sub-sequences for feature analysis, and often fail to capture correlations across distinct series. Moreover, these approaches generally face challenges in generalizing to new categories, leading to potential inaccuracies when analyzing unseen data types. Crucially, they do not incorporate mechanisms for human–machine collaboration, thereby missing opportunities to enhance adaptability and interpretability through interactive feedback and expert insights.

### 2.2. Machine Learning and Deep Learning Approaches for Time Series Analysis

Recent years have seen a paradigm shift in the application of machine learning methodologies to medical time series analysis. Research efforts, as delineated by Soni et al. [43] and Dissanayake et al. [44], have traditionally explored a variety of algorithms—ranging from decision trees and Naïve Bayes to K-nearest neighbors and neural networks—on datasets such as the UCI Cleveland heart disease dataset. Jovic et al. [45] extended this exploration by employing SVM, Ada Boosted C4.5, and random forest algorithms for the classification of cardiac rhythms using time-domain features, while Tripathi et al. [46] introduced a hybrid artificial intelligence framework combining random forest, decision tree, and linear discriminant analysis for insomnia detection. These studies, while pivotal, predominantly hinged on the analysis of static features, overlooking the potential encapsulated within nonlinear latent feature constructions.

In response to this gap, the advent of deep learning techniques has heralded a new era in the analysis of medical time series, particularly accentuating the utility of stacked Recurrent Neural Network (RNN) layers and hybrid RNN configurations for unraveling hidden hierarchical data representations. For instance, Goel et al. [47] and Yazdan et al. [48] leveraged RNNs to enhance the modeling of long-term dependencies, facilitating a more nuanced representation of complex autocorrelation structures. Xu et al. [49] pioneered the design of a tensorized Long Short-Term Memory (LSTM) model to capture sequence-specific historical trends, exemplifying the strides made towards understanding temporal dynamics.

Moreover, the role of deep learning in addressing contemporary health crises and engineering challenges has been underscored by efforts such as those of Chimmula et al. [50], who developed innovative LSTM cell connections for COVID-19 spread prediction, and Siłka et al. [51], who engineered an RNN-LSTM model with hyperbolic tangent activations for predicting high-speed train vibrations.

Additionally, convolutional operations have been adeptly integrated into LSTM architectures to further bolster model efficacy as evidenced by Ullah et al. [52,53], who proposed an end-to-end 2D CNN approach for chronic cardiovascular disease analysis. This methodology, when combined with LSTM [54,55,56] for hybrid ECG signal classification, demonstrates significant improvements in identifying cardiovascular abnormalities by extracting salient features and capturing temporal dependencies.

The incorporation of attention mechanisms represents a substantial leap forward, offering marked enhancements in model interpretability over traditional deep learning paradigms. This advancement, facilitated by the integration of spatio-temporal attention into convolutional recurrent neural networks [57,58,59], has notably improved the interpretability of outcomes. Nonetheless, the automated nature of attention weight training presents challenges in aligning the attention mechanisms with domain-specific expert knowledge.

### 2.3. Prototype Learning Approaches for Time Series Analysis

The paradigm of prototype learning, rooted in case-based reasoning, has been instrumental in delineating representative prototypes to succinctly encapsulate similar instances within observed data or those discerned during model training. Notably, Fu et al. [60] introduced the PEARL methodology, amalgamating prototype with decision rule learning within deep neural network frameworks to enhance both interpretability and the precision of predictive outcomes. Concurrently, the PTAP algorithm [61] harnessed the capabilities of a temporal Convolutional Neural Network (CNN) to distill highly activated periods from activation maps, subsequently classifying these using a meticulously designed prototype selection process. This method’s innovation lies in the formulation of a Gram kernel matrix (elements of a Gram matrix were computed using a kernel function, which effectively measured the similarity or “closeness” between data points in the feature space) predicated on feature values, aiming to rationalize the prototypes derived from time series data.

Further advancements in this field have been marked by Ghods et al. [31] and Tan et al. [62], who employed network architectures that inherently integrate interpretability by concurrently learning a set of prototypes alongside a mechanism to translate these prototypes into visually interpretable representations. Such prototypes, epitomizing learned classes, facilitate an enhanced interpretation of classification results through the lens of similarity with new instances.

To augment the efficacy and resilience of prototype learning, the integration of attention mechanisms [63,64] has emerged as a seminal advancement, enabling focused analysis of pivotal instances and features. Gao et al. [65] notably refined relation classification models within prototypical networks by embedding instances in a support set to compute relation prototypes, thereby facilitating targeted classification. In a similar vein, Lv et al. [66] introduced dynamic prototype units employing attention mechanisms to encode standard dynamics within an auto-encoder structure, where prototypes are aggregated from local encoding vectors weighted by their relevance, culminating in a prototype feature map imbued with task-specific insights through a self-attention-based network [67]. While foundational methodologies have been explored in previous works, some of the research lies in the specialized application and optimization of these methodologies for medical time series data, a domain where such integration has seen limited exploration and application.

Nevertheless, one of the principal challenges in conventional prototype learning is the proliferation of prototypes, which can overwhelm users and obscure the interpretability that is central to the prototype learning approach. This issue often arises from a model’s attempt to capture the diversity within the data, leading to an excessive number of prototypes that dilute their individual significance and make it difficult for end-users, especially those without deep technical expertise, to derive actionable insights. Furthermore, the absence of effective human–machine collaboration restricts the infusion of human intelligence into the modeling process, challenging the potential to harness the complementary capabilities of human intuition and machine computation.

## 3. Materials and Methods

In this section, we first present an overview of the dataset and define the time series classification problem, then describe the architecture and optimization objectives of PahNet in details.

### 3.1. Dataset

The PhysioNet/Computing in Cardiology (CinC) Challenge 2017 dataset [34]: It is designed to foster the development of sophisticated algorithms for the classification of ECG recordings. These recordings, varying in length from 30 s to 60 s, are categorized into four distinct classifications: normal sinus rhythm, atrial fibrillation (AF), alternative rhythm, and recordings deemed too noisy for reliable classification. The dataset comprises 8528 single-lead ECG recordings, all sampled at a frequency of 300 Hz and subsequently band-pass filtered using the AliveCor device. Among these, there are 5154 recordings identified with normal rhythm, 771 with AF rhythm, 2557 classified under other rhythms, and 46 recordings categorized as noisy. This extensive collection of ECG recordings provides a comprehensive resource for validating the efficacy and robustness of classification algorithms in distinguishing among the specified cardiac rhythm categories, thereby contributing significantly to advancements in cardiac health monitoring and diagnosis.

### 3.2. Problem Definition

Given a certain time series $\mathrm{AS}=\{\langle {\left(as_{1}^{\left(t\right)}\right)}_{\left(t=1\right)}^{T},y_{1}\rangle ,\langle {\left(as_{2}^{\left(t\right)}\right)}_{\left(t=1\right)}^{T},y_{2}\rangle ,\cdots ,\langle {\left(as_{N}^{\left(t\right)}\right)}_{\left(t=1\right)}^{T},$ $y_{N}\rangle \}$, where ${\mathrm{as}}_{i}^{\left(t\right)}$∈$R^{n}$ refers to the input time series at a given time step $t\left(t=1,2,\cdots ,T\right)$, *T* denotes the length of the sequence, *y* represents the output label of the input sequence, and *N* stands for the amount of time series. For example, in a health-related application, y may denote the classification result of a target disease, such as the atrial fibrillation. The objective is to train a model to classify or predict the label for any time series $as_{i}=\left\{as_{i}^{1},as_{i}^{2},\cdots ,as_{i}^{T}\right\}$. The used notations are summarized in Table 1.

### 3.3. The Architecture of PahNet

**Figure 1:**
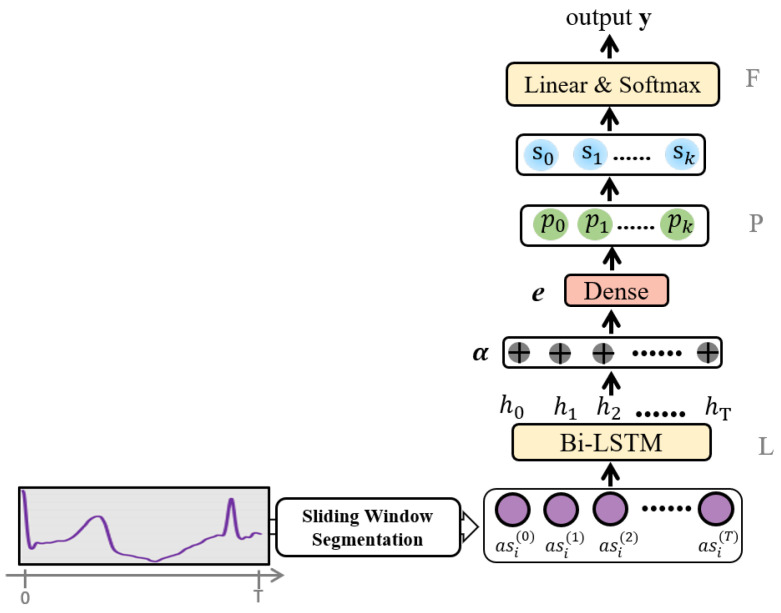
The architecture of PahNe.

The architecture of the proposed model is illustrated in Figure 1, which mainly comprises three components, i.e., a sequence encoder denoted by *L*, a prototype learning layer labeled *P*, and a fully connected layer indicated by *F*.

Given an input sequence ${\left(as_{i}^{\left(t\right)}\right)}_{\left(t=1\right)}^{T}$, the sequence encoder *L* maps the sequence to a fixed-length embedding vector $h=L\left({\left(as_{i}^{\left(t\right)}\right)}_{\left(t=1\right)}^{T}\right)$, $h\in R^{u}$, which represents the sequence as a compact and informative feature representation. Specifically, the encoder can be any standard backbone sequence learning model, such as LSTM, Bidirectional LSTM (Bi-LSTM), or Gated Recurrent Unit (GRU). Additionally, the proposed model includes a long-term attention module that improves the quality of sequence embeddings, converting them to α$h_{i}$$\left(h_{i}=\left\{h_{i}^{1},h_{i}^{2},\cdots ,h_{i}^{T}\right\},h_{i}^{t}\in R^{u},h_{i}\in R^{u\times T}\right)$ and facilitating the identification of optimal time steps (this mechanism, by attributing variable importance to different portions of the sequence, is instrumental in discerning the most significant time steps within the sequence, a capability that directly enhances the fidelity of pattern recognition by ensuring a focus on the most pertinent temporal features). The obtained α$h_{i}$ is then fed into the prototype layer, where it is compared with a set of learnable prototypes ***P*** = {$p_{1},p_{2},\cdots ,p_{k}$} $\left(p_{k}\in R^{u},k=\left\{1,2,\cdots ,K\right\}\right)$, where *K* is the number of prototypes. In such a way, we can perform pattern recognition on time series by comparing the distance between any query time series and the prototypes of various classes. The underlying principle is that the query instance is more similar to prototypes of the same class, compared with the prototypes of other classes.

For the sequence encoder *L*, we employ LSTM to capture long-term dependence in the input time series $as_{i}$. The time series is encoded and mapped into the latent space, and the corresponding process is defined as follows:

(1) $$ig_{t}=\sigma \left(W_{ix}\cdot as_{i}^{t}+W_{ih}\cdot h_{t-1}+b_{i}\right),$$

(2) $$og_{t}=\sigma \left(W_{ox}\cdot as_{i}^{t}+W_{oh}\cdot h_{t-1}+b_{o}\right),$$

(3) $$fg_{t}=\sigma \left(W_{fx}\cdot as_{i}^{t}+W_{fh}\cdot h_{t-1}+b_{f}\right),$$

(4) $$c_{t}=fg_{t}*c_{t-1}+ig_{t}*tanh(\sigma \left(W_{cx}\cdot as_{i}^{t}+W_{ch}\cdot h_{t-1}+b_{c}\right),$$

(5) $$h_{t}=og_{t}*tanh\left(c_{t}\right),$$

where the tensors *W* and *b* are the matrices and bias parameters to be learned during training, $as_{i}^{t}$ is the current input, $c_{t}$ is the cell state vector, and $h_{t}$ is the hidden layer state:

(6) $$\alpha =softmax\left(V_{\alpha }^{T}\left(W_{\alpha }^{T}h_{i}⊙b_{\alpha }\right)\right)$$

(7) $$e_{i}=\sum \alpha h_{i}$$

where $W_{\alpha }\in R^{u\times D_{\alpha }}$ is the weighted matrix at the first layer, $V_{\alpha }\in R^{D_{\alpha }\times 1}$ is the weighted vector at the second layer, ⊙ denotes an addition with broadcasting, $b_{\alpha }\in R^{D_{\alpha }}$, and $e_{i}\in R^{u}$.

The embedding $e_{i}$ is subsequently provided to the prototype layer, based on which a collection of trainable prototypes is obtained. With a distance metric $d_{k}$, the similarity $s_{k}$ between the embedding and a particular prototype is calculated as:

(8) $$d_{k}\left(e_{i},p_{k}\right)={∥e_{i}-p_{k}∥}^{2},$$

(9) $$s_{k}=exp\left(-d\left(e_{i},p_{k}\right)\right),$$

where the function exp(·) converts the distance between the embedding vector $e_{i}$ and the prototype vector $p_{k}$ to the corresponding value of similarity, which ranges from 0 to 1. Afterwards, the fully connected layer applies a linear transformation z=Ws, where $W\in R^{C\times K}$, and *C* denotes the output size, which is equivalent to the number of classes. More details of the proposed model are shown in Algorithm 1.

**Algorithm 1** Classifying time series based on prototype learning and attention mechanisms with the fusion of human–machine intelligence
**Input:**
 Physiological signals *AS*
**Output:**
 The classification result1: *as* = getSeg(***AS***); // split ***AS*** into *T* equal length segments2: *H* = biLSTM(*as*); // convert *as* into features3: α = getAtt(*H*); // calculate the weight of each time step4: $e_{i}$ = sum(α*H*); // output of the LSTM layer5: $P=getPro(p_{1},p_{2},\ldots ,p_{K})$; // refine prototypes based on human–machine intelligence6: $s_{k}=getSim\left(e_{i},p_{k}\right)$; // calculate the similarity metric between $e_{i}$ and $p_{k}$7: z = getFull(***s***); // obtain the classification result

**Figure 2:**
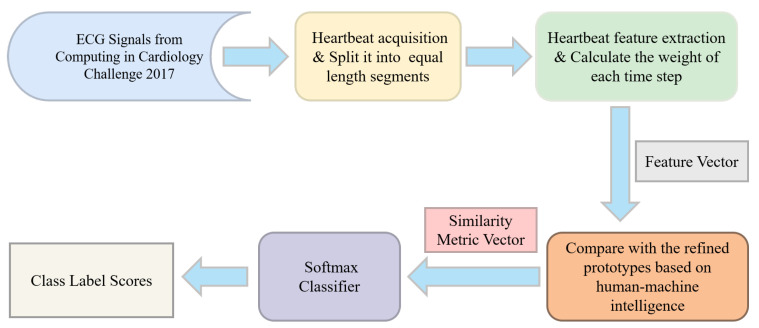
The workflow of the proposed method.

Figure 2 depicts the procedural flow of the methodology proposed.

### 3.4. Optimization Objectives

PahNet’s training objectives comprise three distinct terms, aiming to achieve both high prediction accuracy and good interpretability.

**Diversity**—In the quest to cultivate a diverse and distinctly nonoverlapping set of prototypes within our model, we incorporate a specialized diversity loss term. This term is designed to enforce a minimum mutual distance among the prototypes, thereby enhancing the uniqueness and representativeness of each prototype. Formally, this concept is encapsulated in the diversity loss function, R(P), defined as:

(10) $$R\left(P\right)=\sigma \left(threshold-argmin\left({∥p_{i}-p_{j}∥}_{2}^{2}\right)\right),$$

In this expression, σ(·) denotes the Sigmoid function, serving to scale the loss values between 0 and 1, thus providing a probabilistic interpretation of prototype dispersion. The term threshold signifies a predefined parameter that establishes the criterion for the proximity threshold between any two prototypes. For this model, the threshold value is empirically set to 1, a decision based on experimental evaluations aimed at optimizing the trade-off between diversity and model complexity.

The operational mechanism of *R(P)* ensures that smaller pairwise distances between prototypes $p_{i}$ and $p_{j}$ within the embedding space incur greater losses, compelling the prototypes to maintain a specified minimum distance from each other. This approach not only fosters diversity among the prototypes but also significantly contributes to the overall performance of the model by ensuring that each prototype distinctly captures different aspects of the data representation.

**Prototypicality**—In addressing the challenge of prototypicality, wherein the discrepancy between the encoded instances and their corresponding prototypes may compromise the prototypes’ ability to faithfully represent time series data, our model incorporates a distinct prototypicality loss. This loss function is meticulously designed to ensure that each prototype effectively mirrors the characteristics of at least one instance within the dataset, thereby enhancing the representativeness and accuracy of the prototype representation:

(11) $$\begin{array}{c} R_{1}\left(P,X\right)=\sum_{i=1}^{K}\min_{j\in [1,N]}{∥p_{i}-e_{j}∥}_{2}^{2}, \end{array}$$

where $p_{i}$ denotes the i-th prototype, $e_{j}$ represents the j-th encoded instance, and N signifies the total number of instances. This term aims to minimize the distance between each prototype and its nearest encoded instance, thereby ensuring that prototypes are positioned within close proximity to at least one instance in the dataset.

Conversely, the regularization term $R_{2}\left(P,X\right)$ is articulated as:

(12) $$\begin{array}{c} R_{2}\left(P,X\right)=\sum_{j=1}^{N}\min_{i\in [1,K]}{∥e_{j}-p_{i}∥}_{2}^{2}, \end{array}$$

This term endeavors to cluster similar inputs around each prototype, minimizing the distance between instances and their closest prototype. Collectively, these regularization components not only promote a closer correspondence between prototypes and encoded instances but also facilitate a structured clustering of instances around prototypes, thus bolstering the model’s prototypicality and enhancing its ability to capture the intrinsic patterns within time series data.

**Accuracy**—The accuracy loss component is engineered to refine the fidelity of predictions through the minimization of cross-entropy loss between the forecasted labels and their true counterparts. This critical metric is quantitatively expressed as follows:

(13) $$\begin{array}{c} CE=\sum_{i=1}^{\tilde{N}}y_{i}\log\hat{y_{i}}+\left(1-y_{i}\right)\log\left(1-\hat{y_{i}}\right), \end{array}$$

where $\tilde{N}$ denotes the number of instances within a given mini-batch. Here, $y_{i}$ and $\hat{y_{i}}$ respectively represent the actual and predicted labels for the i-th instance, encapsulating the model’s ability to accurately predict the class of each instance.

Integrating this accuracy loss term with the previously outlined diversity and prototypicality loss components forms the comprehensive optimization objective of our model, articulated as:

(14) $$Loss=CE+\lambda R\left(P\right)+\lambda_{1}R_{1}\left(P,X\right)+\lambda_{2}R_{2}\left(P,X\right),$$

wherein the hyperparameters λ, $\lambda_{1}$, and $\lambda_{2}$ are leveraged to modulate the relative influence of each loss term on the final optimization process. The calibration of these hyperparameters is pivotal, as it necessitates a nuanced understanding of the time series’ inherent characteristics to strike a harmonious balance that optimizes model performance.

### 3.5. Human–Machine Fusion for Responsible Editing

In practical applications, domain experts, such as physicians, need to validate the correctness of machine learning models based on their knowledge. The provision of interpretable models that employ anticipated patterns for predictions is of high importance. To this end, we propose to further refine prototypes in case domain-specific human intelligence is available.

**Figure 3:**
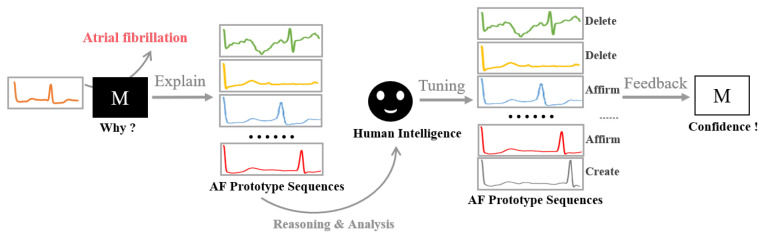
Human–machine collaboration for prototype refinement.

Specifically, human participants, especially domain experts, play a crucial role, as their knowledge and experience offer additional validation and feedback to the trained model. The core concept underlying prototype-based models is the identification of important samples and the assurance of suitable human intervention. Figure 3 illustrates the process of model refining based on human intelligence, aiming to enhance PahNet’s interpretability and performance through the validation and modification of prototypes. By incorporating human expertise, a more collaborative approach is provided, ensuring more trustworthy results for important applications, such as electrocardiogram signal analysis and classification.

According to the knowledge and feedback of domain experts, there are three potential operations that can be applied to the model, including the generation of new prototypes, the validation of pre-existing prototypes, and the removal of current prototypes. Each of these operations serves to improve the model’s overall performance and interpretability. Once a certain operation is conducted, the model is fine-tuned subsequently on the training data to adapt to the newly introduced changes. Such an iterative process fosters a more dynamic and adaptable model, allowing it to better align with domain-specific expertise and real-world applications, such as electrocardiogram signal analysis and classification. In this manner, the proposed model emphasizes the importance of human–machine collaboration by integrating domain expert knowledge for model refinement, which is crucial for its acceptance and adoption by domain experts in fields, where accurate and reliable results are of high importance.

### 3.6. Interpretation with Prototypes

In the proposed model, prototypes are vectors in the latent space, which cannot be interpreted intuitively. To enhance the interpretability of prototypes, it is necessary to transform the prototype vectors back into the original data space. Thereby, we introduce a reverse mapping design during the training process that associates each prototype vector $p_{k}$ with the nearest input sequence in the training set. Such an approach guarantees that each prototype corresponds to an observable and representative time series, which helps improve the model’s interpretability. This is especially important for domain experts, as it enables them to gain a deeper understanding of the model’s decision-making processes, and improve the model based on real-world contexts:

(15) $$\begin{array}{c} \alpha h_{i}\leftarrow \underset{p_{k}\in P}{\arg\min}{∥e_{i}-p_{k}∥}_{2}, \end{array}$$

(16) $$as_{i}\leftarrow \alpha h_{i},$$

In summary, the proposed reverse mapping approach has a positive impact on the performance and interpretability of the model, which facilitates the development of the prototypical technology in practical applications.

## 4. Experimental Results and Discussion

### 4.1. Classification Problem

**Figure 4:**
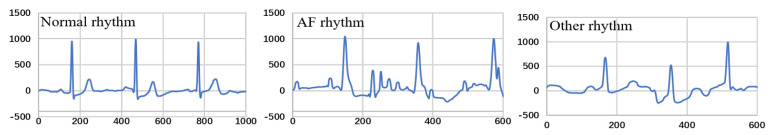
Instances of ECG waveforms.

In this study, we aim to develop an ECG classification model for rhythm identification by categorizing the rhythms as normal sinus rhythm (N), AF (A), or other rhythm (O). ECG waveforms for these three classes in the CinC dataset are depicted in Figure 4. Specifically, the American College of Cardiology (ACC) defines AF as a tachyarrhythmia characterized by predominantly uncoordinated atrial activation resulting in a deterioration of atrial mechanical function [68]. AF is the most common sustained cardiac arrhythmia, which affects 1–2% of the general population [69] and associates with a significant risk of mortality and morbidity, including stroke, hospitalization, heart failure, and coronary artery disease [70]. Despite the importance of AF, its detection remains a challenge due to its episodic nature. Specifically, detecting AF based on a single short lead of ECG is a complex task, and the wide range of rhythm variations makes it even more challenging.

### 4.2. Experimental Setup and Baselines

**Figure 5:**
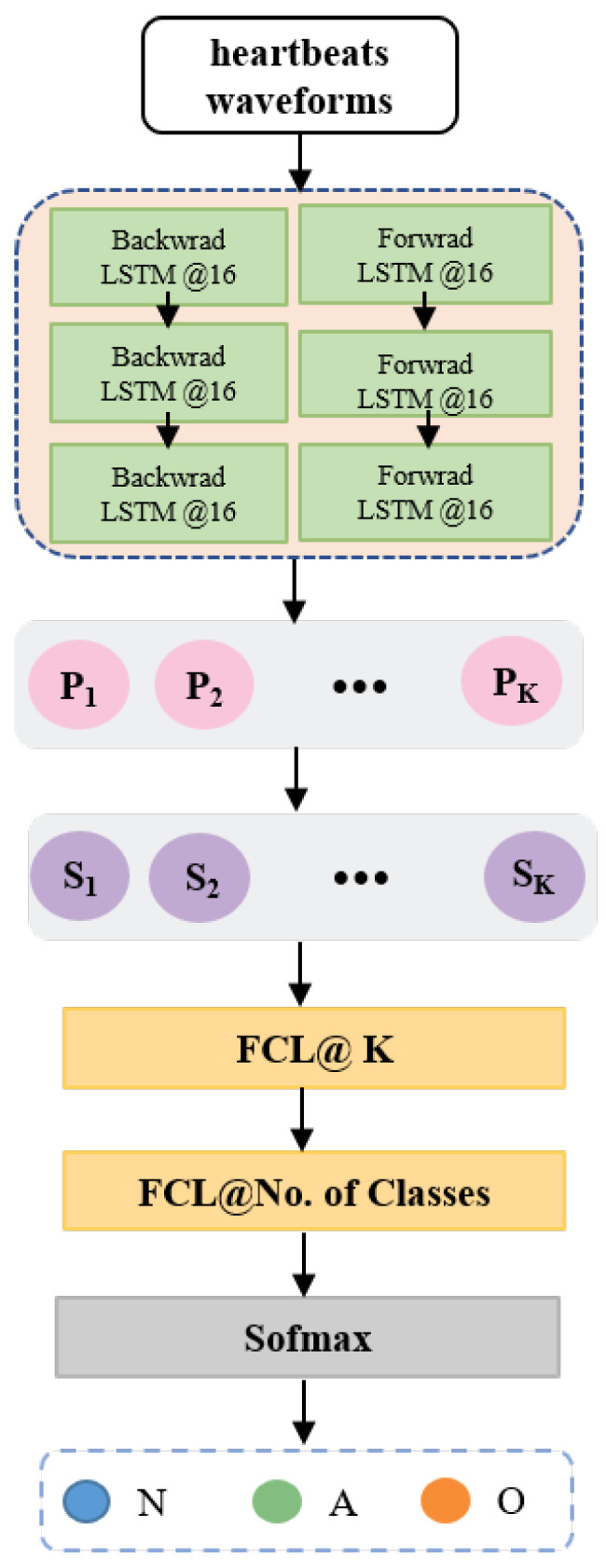
The implementation of PahNet.

*Training and Implementation Details.* To obtain an optimal model, we investigated the performance of bidirectional long short-term memory (Bi-LSTM) networks with varying numbers of layers (ranging from 1 to 8) and hidden units (from 8 to 64), under various configurations involving mini-batch sizes (16, 32 and 128) and optimizers (stochastic gradient descent, adagrad, and Adam). Specifically, a three-layer Bi-LSTM with 16 hidden units was employed, with initial weights/parameters randomly set and learnable parameters updated using the Adam optimizer with a learning rate of 0.002. The fully connected prediction layer employed a dropout rate of 0.1. We set λ = 0.01, $\lambda_{1}$ = 0.05, $\lambda_{2}$ = 0.05, $d_{m}in$ = 2.0. The ECG time series were partitioned into annotated heartbeats according to the protocol proposed in a previous study [71], resulting in 38,363 Atrial Fibrillation samples, 39,480 Normal Sinus Rhythm samples, and 44,571 samples belonging to Other Rhythms. We trained the model on a mini-batch size of 128 samples, where the samples were randomly partitioned into three subsets: a training set (70%), a validation set (10%), and a test set (20%). PyTorch 1.1.0 was used for the implementation of both the proposed model as well as the baselines, and experiments were conducted on a machine with an Intel Xeon E5-2640 processor, 256 GB RAM, 8 Nvidia Titan-X GPUs, and CUDA 8.0 (The supplier is Changzhou Changtao Network Technology Co., Ltd., Changzhou, China). The implementation and workflows of the proposed model are shown in Figure 5.

*Baselines.* To evaluate the performance of the proposed PahNet model, several baseline models are used for comparison in this study.

(a) CNN—The CNN model is applied to the entire ECG segment, followed by a fully connected layer and Softmax layer for classification.

(b) Bi-LSTMAttn—A fusion of the Long Short-Term Memory network and attention mechanisms, which is able to offer interpretability at the level of input variables.

(c) ProSeNet [19]—A model that combines prototype learning with a variant of RNN, which is capable of providing both enhanced interpretability and high accuracy for sequence modeling tasks.

(d) ProtoryNet [27]—A model that operates by identifying the most similar prototype for each sub-sequence within an instance of time series and subsequently feeding a RNN with the corresponding proximity.

### 4.3. Experimental Results

We first conducted a two-class classification task, aiming to differentiate between atrial fibrillation ECG time series and normal ECG time series. To evaluate the models’ performance, various metrics were used, including accuracy (ACC), area under the receiver operating characteristic curve (ROC-AUC), and the F1 score. The results are shown in Table 2. Specifically, we found that the accuracy of nonprototype models outperforms that of prototype models, which is an expected outcome [27]. However, the proposed PahNet model narrows the performance gap in accuracy, which validates the effectiveness of the design.

**Figure 6:**
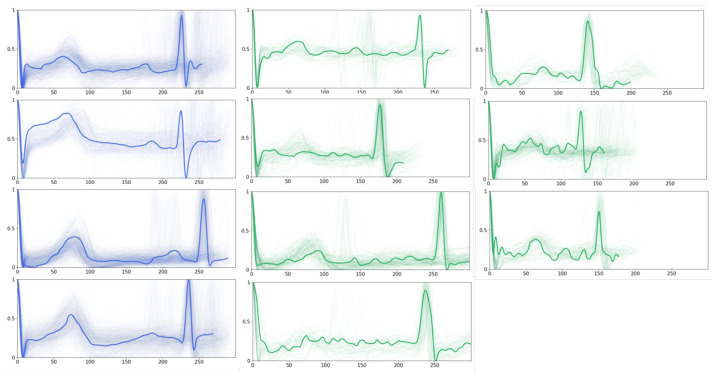
Exemplary prototypes of ECG time series. The blue waveform indicates normal ECG signals, and the green waveform represents AF signals. Transparent lines demonstrate samples within the validation set, which characterize their similarities to the closest prototypes.

Furthermore, we present the obtained prototypes of PahNet in Figure 6, which includes seven ‘AF prototypes’ and four ‘normal prototypes’. In general, the normal P-waves that appear in normal prototypes are superseded by fibrillation waves of varying sizes and shapes in AF prototypes. Moreover, compared with normal ones, AF prototypes display considerable amplitude variations in ventricular waveforms, as well as widening and deforming in the QRS complex. Such observations should be due to the irregular ventricular filling stemming from the erratic atrial electrical activity during ventricular contraction, which leads to amplitude fluctuations. Cardiac experts have validated these observations, which validates the capability of PahNet in learning meaningful prototypes from ECG time series.

We further investigate the model’s performance in classifying three categories, i.e., atrial fibrillation ECG time series, normal ECG time series, and the other ECG time series. The used metrics include accuracy, macro precision, macro recall, and macro F1-score.

The results are summarized in Table 3. Accordingly, we find that compared with the performance in two-class classifications, the models’ performance in three-class classification declines significantly. In particular, the performance of prototype models becomes closer to that of nonprototype models, indicating that models based on prototype learning (e.g., PahNet) can effectively capture subtle differences among AF ECG time series, normal ECG time series, and other ECG time series.

**Figure 7:**
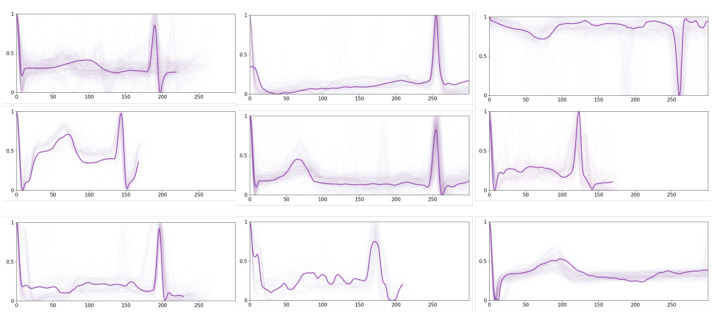
Exemplary prototypes of other ECG time series.

Moreover, we display a set of prototypes for other ECG time series in Figure 7. A broad spectrum of other rhythms can be observed from these prototypes, and some of them exhibit irregular variations. For instance, there are different variations in P-waves, leading to changes in the P-wave morphology, such as widening, heightening, inverting, or even disappearing. There are also abnormalities in the QRS complex, which are reflected by the increased width, atypical shape, or the emergence of supplementary peaks. Such irregularities may signify ventricular arrhythmias or conduction disturbances. Furthermore, deviations in the ST segment are observed, wherein the ST segment may shift upward or downward in relation to the baseline, suggesting potential myocardial ischemia, myocardial injury, or other cardiac complications. Last but not least, there are T-wave abnormalities as well, including asymmetrical height and increased width, or inversion, which may relate to ventricular hypertrophy, electrolyte imbalances, or other cardiac issues.

A comprehensive comparison of the proposed model PahNet and other state-of-the-art prototype-based classification models was conducted, and the results are summarized in Table 2 and Table 3. Accordingly, we can conclude that PahNet achieves the best performance, and the reason should be that these models have distinct architectures.

In the case of ProtoryNet, the input is initially encoded by a sequence encoder before being compared to a set of trainable prototypes in the prototype layer to derive a similarity matrix. Subsequently, the similarity matrix is transformed for classification with the RNN and fully connected layers. As the prototype layer appears in a earlier stage, the need for more refined prototypes arises due to the fact that transformations of the similarity matrix could lead to the loss of valuable information. Experimental outcomes in the original study [27] suggest that ProtoryNet is more suitable for handling relatively longer time series, which explains the significant decline in its performance in comparison to PahNet.

In the case of ProSeNet, it does not incorporate an attention mechanism and solely relies on the outcome of the final step for classification. In contrast, the proposed model integrates an attention mechanism and leverages the outcome of all the steps for classification, which explains PahNet’s performance advantages over ProSeNet.

To sum up, experimental results prove that PahNet achieves accurate and interpretable classification of the ECG time series.

### 4.4. Expert-Guided Prototype Generation

It is easy to understand that prototypes obtained with learning-based models (i.e., machine intelligence) might be incomplete. Therefore, in addressing the challenge of ensuring completeness and accuracy in prototype generation within PahNet, our approach harnesses the synergy of human–machine collaboration to refine prototypes derived through machine learning algorithms. This process begins with the selection of ten volunteers from our research laboratory, who undergo a training session focused on ECG-related knowledge to ensure familiarity with the domain-specific context of the prototypes.

After this orientation, the individuals are divided into five pairs and proceed to a crucial phase of prototype evaluation. An initial step in this phase is to apply a filtering criterion based on the Euclidean distance between ECG signals and the extant prototypes, with signals exhibiting a distance less than a predetermined threshold of two being methodically excluded from further consideration. This filtering ensures that the focus remains on ECG signals that could potentially enhance the nascent prototype set.

These filtered signals are then distributed evenly among the five pairs for a comprehensive initial analysis. Each pair is responsible for identifying signals that are not only emblematic of the broader dataset but also distinct from current prototype representations. Following this identification, domain experts with deep knowledge and experience in the field are called upon to evaluate and affirm the selection of the most representative and distinct signals as new prototypes.

**Figure 8:**
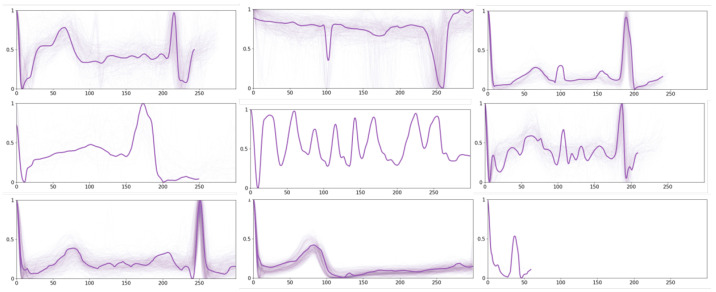
Exemplary prototypes obtained based on human knowledge.

The integration of these expert-validated prototypes into the model involves a comparative analysis with pre-existing prototypes for necessary adjustments and calibration. This is followed by a subsequent round of experimentation, utilizing the refined prototypes now bolstered by expert feedback, to further enhance the prototype representations for additional ECG signals within the model. Specifically, nine expert-selected prototypes are shown in Figure 8.

Moreover, the experimental results of different prototype models that have been refined based on human–machine collaboration are shown in Table 4. By comparing Table 3 and Table 4, we can see that the fusion of human–machine intelligence helps improve the models’ performance.

### 4.5. Results and Discussion

In this manuscript, we unveiled PahNet, a deep sequence model that amalgamates prototype learning with attention mechanisms, targeting the enhancement of both accuracy and interpretability in time series classification. Central to PahNet is a human–machine collaboration mechanism. This design enables domain experts to directly influence and refine the model’s learning outcomes, specifically the prototypes derived from the data. This approach not only makes the model’s decisions more transparent but also more relevant, especially in critical fields such as medical diagnosis, where the precision of data interpretation is paramount.

The experimental results showcased in this paper reveal that our model achieves notable success in binary classification tasks, such as differentiating between normal sinus rhythm and atrial fibrillation. While it exhibits slightly lower performance metrics when compared to certain conventional models, like CNN and Bi-LSTMAttn, it markedly outperforms other leading-edge prototype baseline models. This distinction becomes even more pronounced in triple classification scenarios—encompassing normal sinus rhythm, atrial fibrillation, and other rhythm types—where our model significantly surpasses these baselines, with a prediction accuracy of 0.8414, and macro precision, recall, and F1-score metrics of 0.8449, 0.8224, and 0.8235, respectively.

The results of PahNet illustrates the potential of combining human expertise with machine efficiency to solve complex problems, particularly in healthcare, where it can contribute to more accurate diagnostics, continuous patient monitoring, and the creation of personalized treatment plans. Moreover, the results prompt a broader discussion on the integration of similar models into various application domains, exploring their scalability, adaptability, and impact. The effectiveness of PahNet in engaging domain experts and leveraging their insights for model refinement suggests a promising direction for future research in developing models that are not only technologically advanced but also closely aligned with user needs and practical applicability. As such, PahNet represents a paradigmatic example of how deep learning can be made more accessible and beneficial across disciplines, particularly in those where decision making is deeply intertwined with human expertise.

## 5. Conclusions

This paper introduces PahNet, a deep sequence network that integrates prototype learning with attention mechanisms to enhance the classification of medical time series data, such as ECG signals. Our experiments, utilizing the PhysioNet/Computing in Cardiology (CinC) Challenge 2017 dataset, demonstrate that PahNet successfully balances high accuracy with improved interpretability, particularly excelling in complex triple classification compared to established models.

The distinctive integration of Recurrent Neural Networks with prototype learning in PahNet allows for the effective capturing and dynamic refinement of temporal patterns, which enhances the accuracy of classifying unknown time series samples. The introduction of a human–machine collaboration mechanism further allows domain experts to directly interact with and refine the model’s prototypes, ensuring clinical relevance and enhancing the model’s practical utility.

Moreover, the implementation of a prototype quantity control method manages the number of prototypes, preventing model overcomplexity and maintaining interpretability. This approach addresses common challenges in prototype-based models, particularly the excessive generation of prototypes that can hinder clear decision making.

In conclusion, PahNet represents an advancement in the field of medical time series analysis, offering an interpretable model that holds great promise for real-world clinical applications. Meanwhile, the methodologies developed and validated in this study contribute to the ongoing discussion on strategies for integrating automated systems with human expert knowledge in healthcare settings.

## 6. Limitations and Future Work

The PahNet model still has certain limitations. First, it relies on a single heartbeat rather than a series of heartbeats for ECG classification; therefore, some useful features such as the predictability of the RR interval have not been fully explored. We recognize the importance of RR interval predictability and other features accessible through the analysis of series of heartbeats, both for the depth of analysis they offer and clinical relevance in diagnosing atrial fibrillation or other cardiac conditions. Moving forward, we plan to extend our model to incorporate sequential heartbeat analysis. This expansion will allow us to explore these valuable features more fully and to evaluate their impact on the model’s diagnostic accuracy and predictive capabilities.

Second, the proposed human–machine collaboration mechanism requires further optimization to improve its scalability, especially for large-scale and complex datasets. We plan to improve scalability, which involves a multi-faceted approach, focusing on optimizing the current architecture of our mechanism to handle larger and more complex datasets more efficiently. This will likely include the adoption of more sophisticated machine learning techniques, such as distributed computing frameworks or parallel processing strategies. Additionally, we will explore partnerships with institutions that can provide access to larger and more complex datasets to validate the practical effectiveness of the aforementioned technologies.

Third, in our study, we initially focused on optimizing the collaboration mechanism itself, with a smaller number of experts involved. However, we recognize the significance of this factor and agree that a more extensive exploration into how the quantity of experts affect the model’s performance is essential. To address this, we plan to conduct further experiments that systematically increase the number of experts involved in the process. This will allow us to quantify the impact of the number of experts on the model’s accuracy, reliability, and overall effectiveness.

Fourth, we understand the importance of robust outlier handling and noise mitigation strategies to enhance the accuracy and reliability of models, especially in the complex context of clinical data. While our initial study did not delve into these concerns, we consider them essential components of our future research agenda. In forthcoming work, we intend to dedicate a segment of our research to specifically address these challenges. This will include the integration of advanced various filters to manage outliers effectively such as Adaptive Notch Filter (ANF), the Finite Impulse Response (FIR) filter, and other filter-based approaches.

## Figures and Tables

**Table 1:** Notations.

Notation	Description
**AS**, $as_{i}^{\left(t\right)}$	physiological time series, i-th time series input in **AS** at given time step *t*
***h_i_*** $\in R^{\left(u\times T\right)}$, $h_{i}^{t}\in R^{u}$	i-th time series output of the Bi-LSTM layer, t-th time step in ***h_i_***
***P*** $\in R^{\left(u\times K\right)}$, $p_{k}\in R^{u}$	a set of learnable prototypes, k-th prototype in ***P***
$\alpha \in R^{T}$	the weights of each time step in ***h_i_***
$e_{i}\in R^{u}$	the sum of $\alpha h_{i}$
$d_{k}$	the distance metric of between $e_{i}$ and $p_{k}$
***s***, $s_{k}$	the similar metric between $e_{i}$ and ***P***, k-th similarity in ***s***
*z*, $y_{i}$	the output of fully connected layer, output of i-th classification probability
*L*, *P*, *F*	a sequence encoder, a prototype learning layer, a fully connected layer

**Table 2:** Performance of different models on the two-class classification task.

Models	ACC	ROC-AUC	F1
CNN	0.9280	0.9222	0.9163
Bi-LSTMAttn	0.9324	0.9414	0.9339
ProSeNet	0.8919	0.9047	0.9002
ProtoryNet	0.8731	0.8840	0.8726
PahNet	0.9060	0.9131	0.9177

**Table 3:** Performance of different models in the three-class classification task.

	ACC	Macro-P	Macro-R	Macro-F1
CNN	0.8110	0.8049	0.8224	0.8123
Bi-LSTMAttn	0.8229	0.8137	0.7907	0.8175
ProSeNet	0.8060	0.8209	0.7824	0.7819
ProtoryNet	0.7714	0.7621	0.7836	0.7363
PahNet	0.8101	0.8372	0.8094	0.8104

**Table 4:** Performance of different prototype models on the three-class classification task after incorporating human–machine collaboration.

	ACC	Macro-P	Macro-R	Macro-F1
ProSeNet	0.8376	0.8250	0.8179	0.8163
ProtoryNet	0.8134	0.8013	0.8123	0.8064
PahNet	0.8414	0.8449	0.8224	0.8235

## Data Availability

Data are contained within the article.
